# Isolated Tuberculous Mediastinal Lymphadenopathy in an Immunocompetent Child Without Pulmonary Involvement: A Case Report

**DOI:** 10.7759/cureus.43987

**Published:** 2023-08-23

**Authors:** Sankalp Yadav

**Affiliations:** 1 Medicine, Shri Madan Lal Khurana Chest Clinic, New Delhi, IND

**Keywords:** lymphadenitis, ebus tbna, mediastinal lymphadenopathy, cbnaat/ xpert/ rif assay, mtb (mycobacterium tuberculosis)

## Abstract

Tuberculosis continues to be a big challenge to public health. Isolated cases of tuberculous mediastinal lymphadenopathy in an immunocompetent child without pulmonary involvement are relatively rare. Compared to adults, children are susceptible to such infections; however, due to a lack of specific clinical features and the involvement of invasive techniques in establishing the diagnosis, there are chances of a diagnostic delay. A case of a nine-year-old girl who reported having chest pain for one month is presented. In the absence of the constitutional signs and symptoms of tuberculosis, a diagnosis was confirmed with computed tomography, histopathology, and cartridge-based nucleic acid amplification of samples obtained from endobronchial ultrasound-guided transbronchial needle aspiration. She was put on anti-tuberculous treatment for her weight.

## Introduction

Tuberculosis is a grave issue in endemic settings [1]. It is a noteworthy contributor to morbidity and mortality [2]. The data from India per the Global Tuberculosis Report 2021 states that the incidence and prevalence are 188 and 312 per one lakh (0.1 million) population, respectively [3,4]. A disease that spreads through the inhalation of infected aerosols manifests as pulmonary or extrapulmonary tuberculosis [5]. Pulmonary tuberculosis is when there are bacterial foci in the lungs; however, extrapulmonary tuberculosis (nearly 30%) is a result of the hematogenous or lymphatic spread of bacteria from the lungs to different body parts [6].

Mediastinal lymphadenopathy is a condition due to the enlargement of mediastinal lymph nodes. These enlargements could be a result of an infection like tuberculosis, metastasis, or sarcoidosis (when there is bilateral symmetrical lymph node engagement) [7]. *Mycobacterium tuberculosis* enters the lymphohematogenous system and is lodged in the mediastinal lymph nodes, resulting in swollen nodes [8]. Besides, establishing a diagnosis is challenging, as tuberculosis has been reported to mimic malignancy both radiologically and clinically [8].

A nine-year-old Indian girl presented with chest pain for one month. A detailed history, clinical examination, and serological testing were insignificant. The diagnosis was settled based on the findings of computed tomography, cartridge-based nucleic acid amplification test, and the histopathology of samples from endobronchial ultrasound-guided transbronchial needle aspiration.

## Case presentation

A nine-year-old non-diabetic Indian girl, born of a non-consanguineous marriage, with no developmental delays, was brought in by her parents with complaints of chest pain for one month. She was asymptomatic one month ago when she had pain in her chest localized to the right side. The pain was intermittent and not associated with any aggravating or relieving factors. There was no history of fever, weight loss, cough, or loss of appetite. There was no history of trauma or falls, and there was no history of tuberculosis in her or any contacts. She was fully vaccinated for her age, and there was no history of migration or stays at refugee camps or night shelters.

A general examination was suggestive of a thin-built girl with normal vital signs. Her systemic examination was insignificant in terms of any major issues. Further, there was no icterus, cyanosis, clubbing, edema, or lymphadenopathy. Her laboratory tests are shown in Table 1.

**Table 1:** Diagnostic workup of the patient HGB: Hemoglobin; MCH: Mean Corpuscular Hemoglobin; MCHC: Mean Corpuscular Hemoglobin Concentration; MCV: Mean Corpuscular Volume; PCV: Packed cell volume; RDW: Red Cell Distribution Width; RBC: Red Blood Cell; WBC: White Blood Cell; DLC: Differential Leukocyte Count; ESR: Erythrocyte Sedimentation Rate; ALK PHOS: Alkaline Phosphatase; AST: Aspartate Aminotransferase; ALT: Alanine Aminotransferase; HCV: Hepatitis C Virus; USG: Ultrasonography

Investigation	Results	Reference range
HGB	10.9	11.5-16.0 g/dL
MCH	30.6	27-33 pcg
MCHC	32.0	31-36 g/dL
MCV	92.0	85-100 fl
PCV	41.0	38.3% to 48.6%
RDW	13.0	0-14%
RBC	4.8	4.7 to 6.1 million cells/mcL
WBC	7.1	4.5-12.0 K/uL
DLC		
Neutrophils	64	55-70%
Lymphocytes	26	20-40%
Monocytes	8	2-8%
Eosinophils	1	1-4%
Basophils	1	0-1%
ESR	50.0	0 to 22 mm/hr
Serum sodium	135.0	135-145 mmol/L
Serum potassium	4.64	3.5-5.1 mmol/L
Serum calcium	8.2	8.5-10.5mmol/L
Serum chloride	99.0	98-107 mmol/L
Blood culture	Sterile	Sterile
Serum bilirubin (total)	0.60	0.2-1.0 mg/dL
Serum bilirubin (direct)	0.35	0.2-1.0 mg/dL
Serum bilirubin (indirect)	0.25	0.2-1.0 mg/dL
ALK PHOS	110.0	30-115u/L
Albumin	3.5	3.5-5 g/dl
Serum creatinine	0.59	0.51-0.95 mg/dL
AST	29.0	0-40u/L
ALT	33.0	0-40u/L
Anti-HCV antibodies	Non-reactive	Reactive-Non-reactive
HIV (I and II)	Non-reactive	Reactive-Non-reactive
Fasting blood sugar	90.0	70-99 mg/dL
Activated partial thromboplastin time	33	25-35 seconds
Alpha-fetoproteins	10	10–20 μg/L
Throat culture	Sterile	Sterile
Serum angiotensin-converting enzyme levels	30	<40 nmol/mL/min.
C-reactive proteins	0.3	0.3 to 1.0 mg/dL
Bone marrow biopsy	Unremarkable	Unremarkable-Remarkable
Mantoux test	21	0-15 millimetres
Echocardiography	Unremarkable	Unremarkable-Remarkable
Electrocardiogram	Unremarkable	Unremarkable-Remarkable
USG-whole abdomen	Unremarkable	Unremarkable-Remarkable

**Figure 1:**
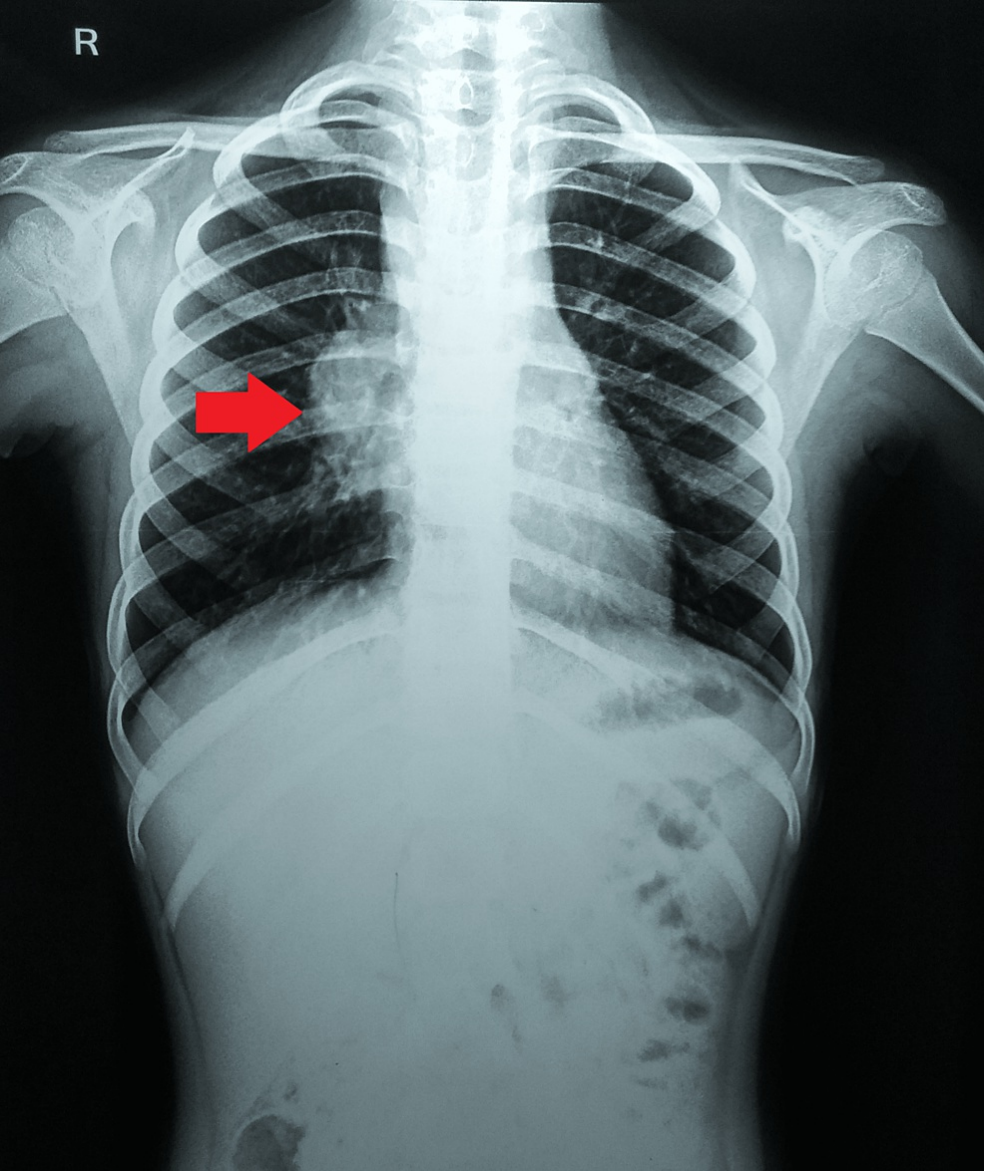
Plain chest radiograph posteroanterior view Arrow showing right hilar lymphadenopathy

A chest radiograph was suggestive of right hilar lymphadenopathy with mediastinal widening (Figure 1).

**Figure 2:**
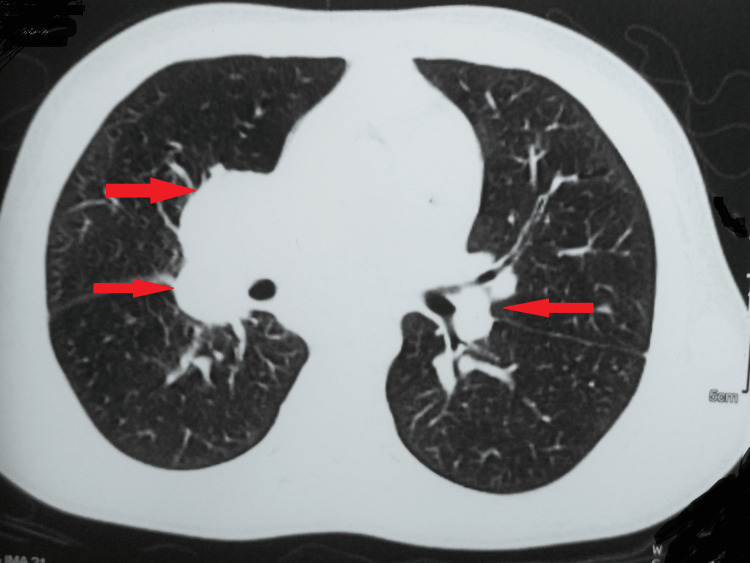
Computed tomography of the chest Arrows showing multiple enlarged conglomerated necrotic lymph nodes in the mediastinum

**Figure 3:**
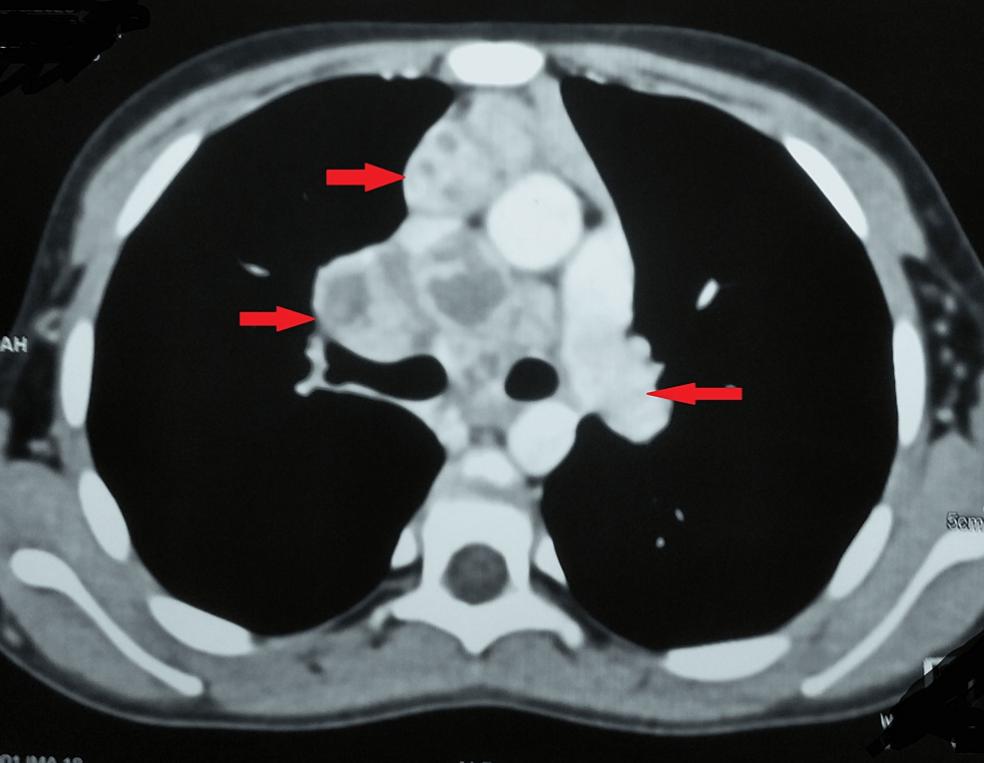
Computed tomography of the chest Arrows showing several enlarged conglomerated necrotic lymph nodes in the mediastinum

She underwent an induced sputum microscopy for acid-fast bacillus and a cartridge-based nucleic acid amplification test of bronchoalveolar lavage. However, both results were negative. Her computed tomography of the chest was remarkable for multiple enlarged conglomerated necrotic lymph nodes in the mediastinum at the paratracheal, precarinal, pretracheal, subcarinal, prevascular, and right hilar regions (Figure 2 and Figure 3).

To establish the diagnosis, an endobronchial ultrasound-guided transbronchial needle aspiration was performed, as the patient’s family refused a transthoracic biopsy. The samples obtained were sent for cytopathological and bacteriological examination, a cartridge-based nucleic acid amplification test/GeneXpert, a line-probe assay, and culture. The typical histological findings were suggestive of tuberculosis (grade I pathologic result: granulomatous reaction with caseation necrosis). The cartridge-based nucleic acid amplification test/GeneXpert detected *Mycobacterium tuberculosis *(low) with no resistance to rifampicin. The results of the line-probe assay and culture were negative.

So, a final diagnosis of isolated tuberculous mediastinal lymphadenopathy in an immunocompetent child without pulmonary involvement was made, and she was initiated on anti-tubercular chemotherapy as per the national guidelines with fixed-dose combinations with details as mentioned in Table 2.

**Table 2:** Anti-tubercular chemotherapy for six months

Phase	Drug	Dose	Duration
Intensive phase	Rifampicin	10 mg/kg	8 weeks
	Pyrazinamide	25 mg/kg	8 weeks
	Ethambutol	15 mg/kg	8 weeks
	Isoniazid	5 mg/kg	8 weeks
Continuation phase	Rifampicin	10 mg/kg	16 weeks
	Ethambutol	15 mg/kg	16 weeks
	Isoniazid	5 mg/kg	16 weeks

Additionally, a tablet of pyridoxine (1 mg/kg) was also added. Currently, she has completed 20 days of her treatment and has not reported any adverse drug reactions. She was also given counseling for treatment adherence and a healthy diet with maintenance of hygiene.

## Discussion

Lymphadenitis is a significant extrapulmonary manifestation of tuberculosis [9]. Tuberculous lymphadenitis contributes about 4-7% of the total tuberculosis load, with mediastinal lymphadenopathy making up about 10% of all these cases [9]. It has a proclivity for young females and children, with 20-40 years as the peak age of onset [9]. The cervical lymph nodes are the most common sites for tuberculosis; however, the mediastinal, axillary, and inguinal lymph nodes are also found to be associated with the infection [10].

The findings of mediastinal mass are a common clinical scenario in outpatient departments [7]. The diagnosis is challenging, as a number of structures lie in this anatomical area, including the lymph nodes, thymus, nerves, vessels, adipose tissue, and occasionally the thyroid gland [11]. The commonest causes of a mediastinal mass are teratoma, thyroid disease, thymoma, lymphoma, and enlarged lymph nodes due to infection, metastatic, or sarcoidosis [7]. After its entry into the respiratory system, *Mycobacterium tuberculosis* undergoes lymphohematogenous dissemination [8]. Usually, the mediastinal and hilar lymph nodes are the earliest lymphatic tissues that the bacteria will run into [8].

Diagnosis is often delayed, as the patients are mostly asymptomatic [8]. Patients become symptomatic when there is a mass effect due to enlarged lymph nodes that compress the adjacent structures, resulting in chest pain, dyspnea, or coughing [8]. A definite diagnosis is achieved after a detailed history, clinical examination, lab investigations (including tumor markers), and Gene Xpert analysis [8]. Further, the diagnosis and extent of involvement of the mediastinum can be determined by contrast computed tomography of the thorax [8]. Histopathological confirmation is essential before any therapeutic intervention [8]. Furthermore, as seen in the present case, samples for the same could be obtained by bronchoscopy or endoscopic bronchial ultrasound, allowing for fine-needle aspirate cytology or transbronchial needle aspiration [8,12]. These techniques have been found to be safe for children and can be undertaken with moderate sedation [12].

The mainstay of treatment is medical management with anti-tuberculous drugs [9]. Surgery is indicated only in extreme cases when the vital structures are compressed by the mass [9]. If not treated promptly, mediastinal lymphadenitis could end up with dysphagia due to extrinsic compression, vocal cord palsy resulting in recurrent laryngeal nerve palsy, or fistulation extending into the esophageal wall [9].

In a study by Alves et al. on 99 subjects with a mean age of 13.5 years, mediastinal lymphadenopathy was reported in 81% with a maximum diameter of 7 mm on the smallest axis [13].

A case similar to the present case was published by Kathwate in the year 2022 in an eight-year-old girl [14]. However, the present case differs from his in the absence of axillary abscess, pericardial and bilateral pleural effusion, and ascites. Moreover, there was no aortic encasement in the present case [14].

In short, the case of a nine-year-old girl was presented here. Although mediastinal lymphadenitis is common in children, this case stresses the importance of diagnosis, especially in the absence of constitutional signs of tuberculosis. A lack of constitutional signs and symptoms could result in a diagnostic delay with unfavorable outcomes.

## Conclusions

The case of a nine-year-old Indian girl with intermittent chest pain for one month is presented. A detailed history, clinical examination, and extensive laboratory workup were futile. The diagnosis was achieved based on the findings of computed tomography with a cartridge-based nucleic acid amplification test and the histopathology of samples obtained by endobronchial ultrasound-guided transbronchial needle aspiration. She was also initiated on anti-tubercular therapy. A high index of suspicion is essential for the timely diagnosis and the initiation of management in such cases.
